# Creating infinite contrast in fluorescence microscopy by using lanthanide centered emission

**DOI:** 10.1371/journal.pone.0189529

**Published:** 2017-12-13

**Authors:** Miguel R. Carro-Temboury, Riikka Arppe, Casper Hempel, Tom Vosch, Thomas Just Sørensen

**Affiliations:** 1 Nano-Science Center & Department of Chemistry, University of Copenhagen, Copenhagen, Denmark; 2 Department of Micro- and Nanotechnology, Technical University of Denmark, Kgs Lyngby, Denmark; Oregon State University, UNITED STATES

## Abstract

The popularity of fluorescence microscopy arises from the inherent mode of action, where the fluorescence emission from probes is used to visualize selected features on a presumed dark background. However, the background is rarely truly dark, and image processing and analysis is needed to enhance the fluorescent signal that is ascribed to the selected feature. The image acquisition is facilitated by using considerable illumination, bright probes at a relatively high concentration in order to make the fluorescent signal significantly more intense than the background signal. Here, we present two methods for completely removing the background signal in spectrally resolved fluorescence microscopy. The methodology is applicable for all probes with narrow and well-defined emission bands (Full width half-maximum < 20 nm). Here, we use two lanthanide based probes exploiting the narrow emission lines of europium(III) and terbium(III) ions. We used a model system with zeolites doped with lanthanides immobilized in a polymer stained with several fluorescent dyes regularly used in bioimaging. After smoothing the spectral data recorded in each pixel, they are differentiated. Method I is based on the direct sum of the gradient, while method II resolves the fluorescent signal by subtracting a background calculated via the gradient. Both methods improve signal-to-background ratio significantly and we suggest that spectral imaging of lanthanide-centered emission can be used as a tool to obtain absolute contrast in bioimaging.

## Introduction

Fluorescence microscopy relies on the ability to detect the difference between a signal emitted from a probe and the background [1–6]. This can be done by using a probe emitting a strong signal or a method that exploits probes emitting a unique signal [7–13]. The methods we demonstrate here are able to fully remove the background, thereby allowing for unique identification of fluorescent signals.

We recently introduced a model system that we suggest as a benchmark for developing new microscopy methods [14]. In our model system we use lanthanide ions as the signal that is to be discriminated from a background simulated by emission from bright organic fluorophores, see Methods and Materials for details. While lanthanide centered emission has been studied extensively in time-gated imaging as well as in near infra-red (NIR) imaging [13, 15–20], lanthanide based probes have mainly found use in time-resolved Förster Resonance Energy Transfer (FRET) based high-content screening and in the DELFIA assay [21–34]. This may be due to the fact that microscopes capable of time-gated measurement of emission in the millisecond range are rare. Here, we propose a method of exploiting the unique properties of lanthanide centered emission in spectral imaging without time-gating [35].

We demonstrate two different data treatment methods that analyze spectrally resolved imaging data by exploiting the narrow emission bands arising in the *f*-*f* transitions of europium(III) and terbium(III) ions. The most successful of the methods exclusively identifies the sharp emission lines arising from lanthanide centered emission, and automatically subtracts the background signal while maintaining the fluorescent signal originating from the luminescent probes resulting in infinite contrast. Previously, background subtraction methods have been used in Raman imaging [36], and for specific and general image analysis [37, 38]. However, we chose to develop our own dedicated spectral imaging tool based on gradient analysis and thresholding designed for lanthanide based imaging. As a result, we can construct fluorescent images where the origin of the photons used to generate an image can be assigned with absolute certainty. In general terms, image contrast is defined as the brightest recorded signal in the image divided by the dimmest recorded signal. Basic image processing without the algorithm we present could maximize the image contrast by setting the dimmest signal to zero and thus achieve infinite contrast. However, this contrast would not be lanthanide specific as it would include the contribution from the background signal and from other fluorescent probes. The algorithm presented here provides lanthanide luminescence specific images with infinite contrast.

## Methods and materials

The methods here described are reiterated and adapted from ref. [14]. Fluorescein F18 was from (Sigma-Aldrich, St. Louis, MO, US), MitoTracker Red (MT, CMXRos) from Molecular Probes (ThermoFisher Scientific, Waltham, MA) and ATTO647N from ATTO-TEC GmbH (Siegen, Germany). Tb(III) acetate hydrate (99.9%), and Eu(III) acetate hydrate (99.9%) were purchased from Sigma-Aldrich (St. Louis, MO). Linde Type 5A (LTA) zeolites were a gift from UOP Antwerpen. Poly(vinyl alcohol) (PVA, 98% hydrolyzed, average M_w_ 13 000–23 000) was from Sigma-Aldrich.

### Model system

Our model system consisted of lanthanide(III) ions doped in zeolites and a polymer thin film dyed with fluorophores. The Ca^2+^ cations inside the pores and cavities of Linde Type A (LTA) zeolite were first exchanged with Eu(III) or Tb(III) cations by mixing 200 mg of zeolite in 800 μl of 0.25 M Ln(III) acetate hydrate in milliQ-water over night at room temperature (Vortex 3, IKA, Staufen, Germany). The Ln(III) exchanged zeolites were recovered and washed with 1 ml of milliQ-water thrice by centrifugation (1000 rpm, 2 min, Force 12, Denver Instrument, Bohemia, NY) and finally dispersed into 1 ml of MQ-water. The polymer thin film was formed by mixing 2 mg of Eu(III)@LTA and 2 mg of Tb(III)@LTA with 3% (w/v) PVA dyed with 150 μM of F18 (5-octadecanoyl amino-fluorescein), 0.1 μM of MitoTracker Red (MT), and 0.1 μM ATTO647N (only for the sample with Eu(III)@LTA), and 50 μl of this mixture was spin-coated (SCI-10, Novocontrol, Montabaur, Germany) using dynamic dispense for ~1 min at ≈4000 rpm on a 22 × 22 mm microscope cover slip (Menzel-Gläser #1.5). Prior to use, the microscope slides were cleaned by pyrolysis at 450°C for a minimum of 1 hour. The samples used in this manuscript are the same as in ref [14].

### Confocal fluorescence microscope

A SuperK EXTREME EXB-6 supercontinuum white light laser with a SuperK SELECT wavelength selector (NKT Photonics, Birkerød, Denmark) was used as the excitation source. Two excitation wavelengths were selected: 465 nm for Eu(III), and 488 nm for Tb(III) and F18. The laser powers with 77.88 MHz repetition rate for each wavelength were 2 μW and 7.2 μW, respectively. A shortpass filter (540AESP, Omega optical) was added to the excitation light path and long pass filters (2x 532LP, BLP-01-532R-25, Semrock) to the emission light path to ensure clean excitation lines and to exclude scattered excitation radiation from the emission window, respectively.

The home-built scanning fluorescence confocal microscopy setup was based on an Olympus IX71 inverted microscope with a piezo-driven scanning stage (P5173CL, Physik Intrumente, Karlsruhe, Germany), controlled by a home-written software program (LabView, National Instruments), allowing for point-by-point imaging of the sample in a raster scanning fashion in a range up to 100 μm × 100 μm. Upon laser illumination, the emission signal from the sample was collected by the same 100× oil immersion objective (Olympus UPLFLN 100×, 1.3 NA). A 70/30 beamsplitter (XF122, Omega Filters) was used in the microscope instead of a dichroic mirror.

The emission light was focused through a 50 μm pinhole, directed through optical long pass filters (see above) and detected in a CCD-based spectrometer (Princeton Instruments SPEC-10:100B/LN_eXcelon CCD camera, SP 2356 spectrometer with 1-030-500 grating 300 g/mm @ 500 nm, all controlled by the same LabView program that controls the scanner). The X axis of the emission spectra was calibrated using emission lines of a neon lamp (6032 neon lamp, Newport Corporation, Irvine, CA). The Y axis (Intensity) was not corrected for differences in optical transmission and detection efficiency.

### Data collection and analysis

From the dyed polymer thin film a single zeolite was located for imaging. The zeolite and the dyed PVA-film surrounding were imaged so that each excitation wavelength applied to the same sample was used in separate corners of the zeolite by placing the center of the zeolite in one corner of the image. This minimized the bleaching of the dyes. An area of 5 μm×5 μm with 10 × 10 pixels was imaged with 1 s integration time per pixel, and the emission spectrum following the excitation at a given excitation wavelength was recorded for each pixel. The images were created and analyzed with a home-written Matlab® (MathWorks, Natick, MA) routine. The signal from the lanthanides is located at the edges of the zeolite, where it overlaps with the signal from the organic dyes. The background subtraction was performed using a home-written Matlab® routine described in the following section. The results were compared with those obtained with the built-in function *msbackadj* from Matlab® software, which estimates the baseline in multiple shifted windows. Total intensity images were formed by integrating the spectra over the whole spectral range.

### Description of gradient based background subtraction algorithms

Method I reports the sum of the absolute value of the gradient as a probe of fluorescent signals arising from sharp emission lines, while Method II completely resolves the fluorescent signal arising from the sharp emission lines.

#### Method I

This method is used to detect sharp emission in a faster and simpler manner, by using the absolute value of the gradient of the smoothed data. It can offer a quick assessment of the location of sharp features in an image.

1. The raw spectrum *S’* is smoothed giving the spectrum *S*
  - Savitzky-Golay smoothing is performed to ensure that the gradient arises from the specific signal
  - The vector with the raw data (S) has 1340 entries, from 1340 pixels in the CCD.
2. The gradient is calculated with *Δy*/*Δx* in *Δcounts*/*Δpixel*.
  - The pixel increment is proportional to wavelength increment (as a very good approximation), and *Δpixel* = 1 (The gradient can be also calculated in wavenumber; however the performance is very similar using the data presented here).
3. The *Δcounts*/*Δpixel* gradient is calculated for each position in the vector
  - Each gradient matches a pixel number.
4. The gradient is smoothed (Savitzky-Golay), giving the gradient signal *Δ*.
  - Example of a gradient corresponding to a peak followed by a positive slope: *gradient* = [1 2 4 6 8 6 2–4–6–8–7–3–1 3 6 8 10 …]
5. The absolute value of the gradient is calculated, giving the signal *L(I)*
6. The signal can be integrated in the regions of interest to form the images based on gradient.
7. Optionally, cosmic rays can be discarded by using an upper limit on the gradient, since they are usually extremely sharp and bright

#### Method II

This method finds anchor points placed at the onset of high absolute value gradient regions (beginning and end of slopes of the signal *S*), defined by *T1*. The anchor points can mark the beginning of the slope (“onset anchor”) or the end of the slope (“end anchor”). If two end–onset anchors are too close to each other (threshold *T2*), these anchor points are discarded. In this way only the onset and end of a sharp band are pinned and consecutive fine structure features are ignored. Afterwards, the anchor points are used to define a baseline *B*—the contribution of the F18 dye background- that is used to extract the lanthanide contribution *L(II)*. The points in between consecutive onset–end anchor points are linearly interpolated to have *B* with the same number of points as the original spectra *S*. For the points in between consecutive end–onset anchor points, *B = S* is made. At the end of the process, the lanthanide spectra *L* is found as *L = max(S–B*, *0)*. Steps 1–4 are the same as in method I, the new steps start from 5 in resolving the signal of a given spectrum:

- The sharp features are discriminated using a gradient threshold T1.

Too low threshold converts noise into false features, while too high threshold would not capture the band features. For the integration time of 1 s used here, adequate for the brightness of the probes, a value of *T1* = 5 is a good compromise and robust value. For very bright signals the threshold needs to increase following the increased photon noise that scales as the square root of the signal.

5 A vector *d1* is defined where the points of the gradient with a value greater than *T1* are assigned a value of 1 and the points with a value lower than -*T1* are assigned a value of -1. All the other values are made 0.
  - Following the example: *d1* = [0 0 0 1 1 1 0 0–1–1–1 0 0 0 1 1 1…]
6 A vector *d2* is defined where the 0s next to a 1 or -1 are assigned a value of 2.
  - Following the example: *d2* = [0 0 2 1 1 1 2 2–1–1–1 2 0 2 1 1 1…]
7 A vector *d3* is defined as *d3* = *d2(i+1)*-*d2(i)* and an entry with value 0 is added at the beginning.
  - Following the example: *d3* = [0 0 2–1 0 0 1 0–3 0 0 3–2 2–1 0 0…].
8 The values of *d3* that are different from 2 or -2 are deleted, leaving only the beginning and end of feature (slope) information.
  - In the example *d3* = [0 0 2 0 0 0 0 0 0 0 0 0–2 2 0 0 0…].
9 The value 2 represents a start of feature and the -2 represents an end of feature. Thus, the positive or negative sign means onset or end, respectively.
  - The vector d3 contains the information on the position of all the preliminary anchor points.
10 An error finding algorithm is run to remove consecutive equal non-zero elements in the vector. From consecutive -2 values only the rightmost is kept, and from consecutive 2 values only the leftmost is kept.
  - Consecutive start/end of features may appear in some instances.
11 The distance in wavenumber between the end of feature and the next beginning of feature is calculated.
12 If the distance is below a threshold *T2* the two anchors are eliminated. For the vibrational sub-bands of the lanthanide probes used, a value of *T2* = 200 cm^-1^ is adequate.
  - This is done to remove anchors at the peak maxima and in between vibrational sub-bands.
  - The only feature left in this example is at the position 3 of the vector d3, *d3* = [0 0 2 0 0 0 0 0 0 0 0 0 0 0 0 0 0…].
13 A vector “Anchor” containing all the actual anchor positions in the vector *d3* is created. The positions of the first (1) and last (1340) CCD pixels are added as “end” and “onset” anchors respectively.
  - From the example, Anchor = [1 3 …1340]. The number 3 comes from *d3*(3) = 2.
  - The information of the sign (onset or end) of an anchor position is kept in d3
  - The anchors stored in the vector Anchor have alternating sign, i.e. are ordered as [end0 onset1 end1 onset2 end2 …onset0]. The zero label indicates the added anchors at the first and last positions and the non-zero labels indicate features such as bands or the filter feature.
14 The second and third anchor points of the Anchor vector are checked to distinguish emission onset from the filter onset where emission occurs close to the filter onset. A filter feature is defined with a threshold T3. This threshold is a specific requirement due to our combination of filters, but is not needed in general, if a band does not overlap with the filter onset. In our case the value T3 = 40 identifies properly the filter onset.
  - If the start and end of feature are closer than *T3*, the feature is the filter onset and is discarded. That is if *Anchor*(3)-*Anchor*(2)<*T3*, then the two anchors are removed from the Anchor vector. This will ensure *L* = 0 until the first lanthanide band.
  - In the case of an early onset of signal *L*, the filter onset is the start of feature and the end of the first emission band is the end of feature. Then the program moves *Anchor(2)* to the estimated onset of the band. That is the point before the maximum of such band, where the signal *S* is closer to *S(Anchor*(3)) located at the end of the band. In other words, for the sake of simplicity the baseline for this specific band is made a line as close to horizontal as possible.
  - The latter is practically implemented by creating a new vector *P = S*-*S(Anchor*(3)) and finding the minimum pixel value *p*_*min*_ greater than zero. Then *Anchor(2) = p*_*min*_
15 The baseline or background *B* is defined by linearly interpolating the values between consecutive onset–end anchors, and by making equal to *S* the points between consecutive end–onset anchors. More precisely,
  $$B=\left\{ \begin{array}{l} \;S,\;Anchor(2n-1)\leq pixel\leq Anchor(2n)\\ \mathrm{Lin}(Anchor(n),Anchor(2n)),\;Anchor(2n)<pixel<Anchor(2n+1) \end{array}\right.$$
  Where *n = 1*,*2…* and *Lin* indicates linear interpolation between the two values indicated.
16 The baseline is subtracted from the raw data: *L* = *S–B*
17 Values of *L* below zero are set to zero after subtraction of the baseline: *L = max(S–B*, *0)*
  - The spectra *L* are set to zero if the total counts are below a threshold *T4* = 2000. (Optional). This removes part of the cosmic rays. Another optional threshold *cosmicthres = 500* removes cosmic rays using a gradient threshold higher than T1. (Optional)
18 The resolved spectrum *L* and the background *B* are plotted.

The Matlab® software scripts are available as supporting information. The script *SpectralImaging_SharpBands_InfiniteContrast*.*m* is a function called from the main program that contains the main program displaying the images and spectra, the script *AutoBackgroundRemove_2p3*.*m* contains the code to generate the spectrally resolved signals L(II), L(I), S and X. The Printer_subplot_Mig.m is a function called from the main program that contains some auxiliary code for plotting figures. All these files should be stored in the same folder.

## Results

### Data treatment methods I and II

**Fig 1:**
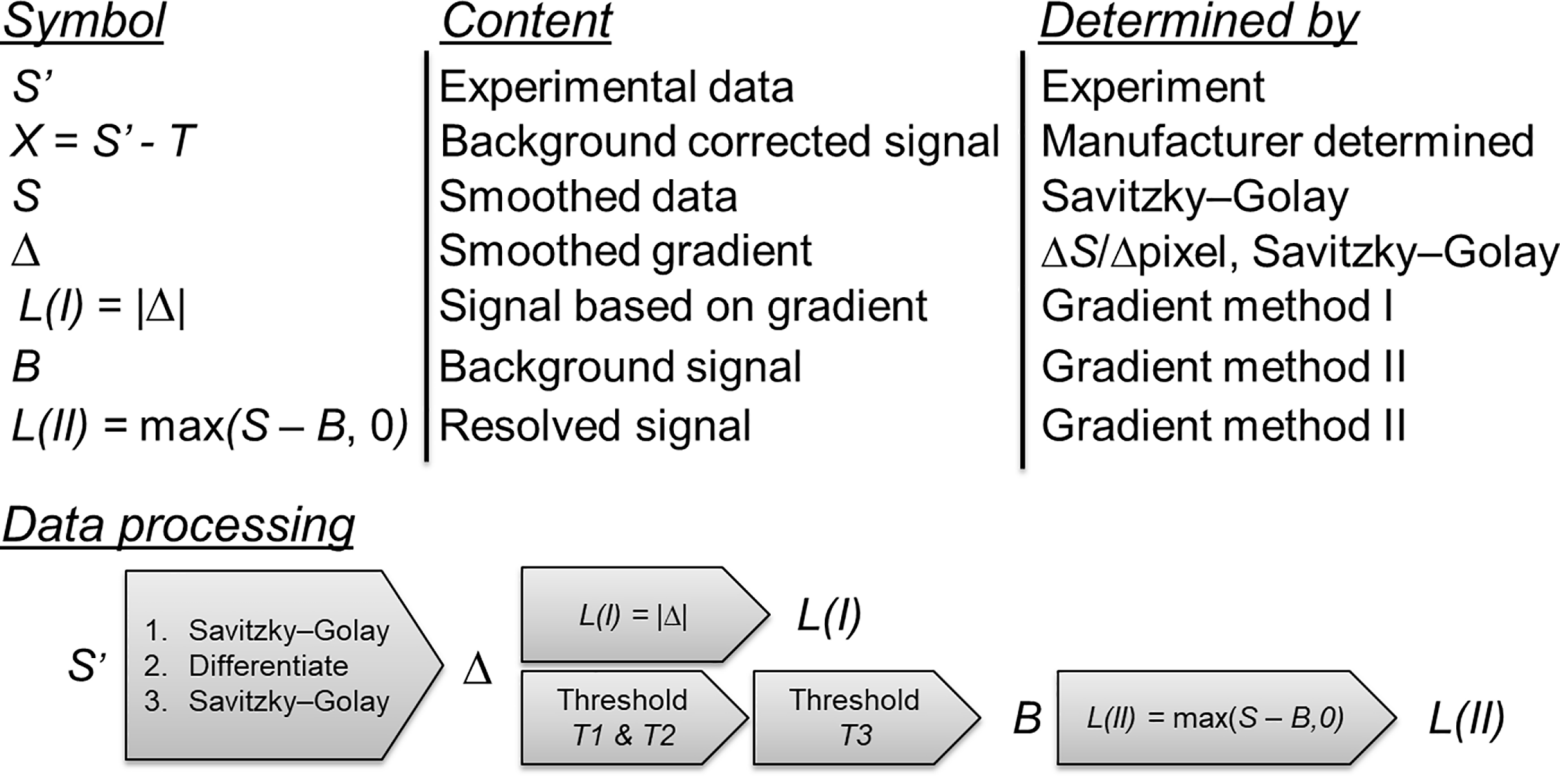
Definition of terms and description of data treatment methods I and II. Top: Definitions of symbols describing data arrays, content of arrays, and the method of determination. Bottom: schematic description of data processing leading to the resolved signals.

**Fig 2:**
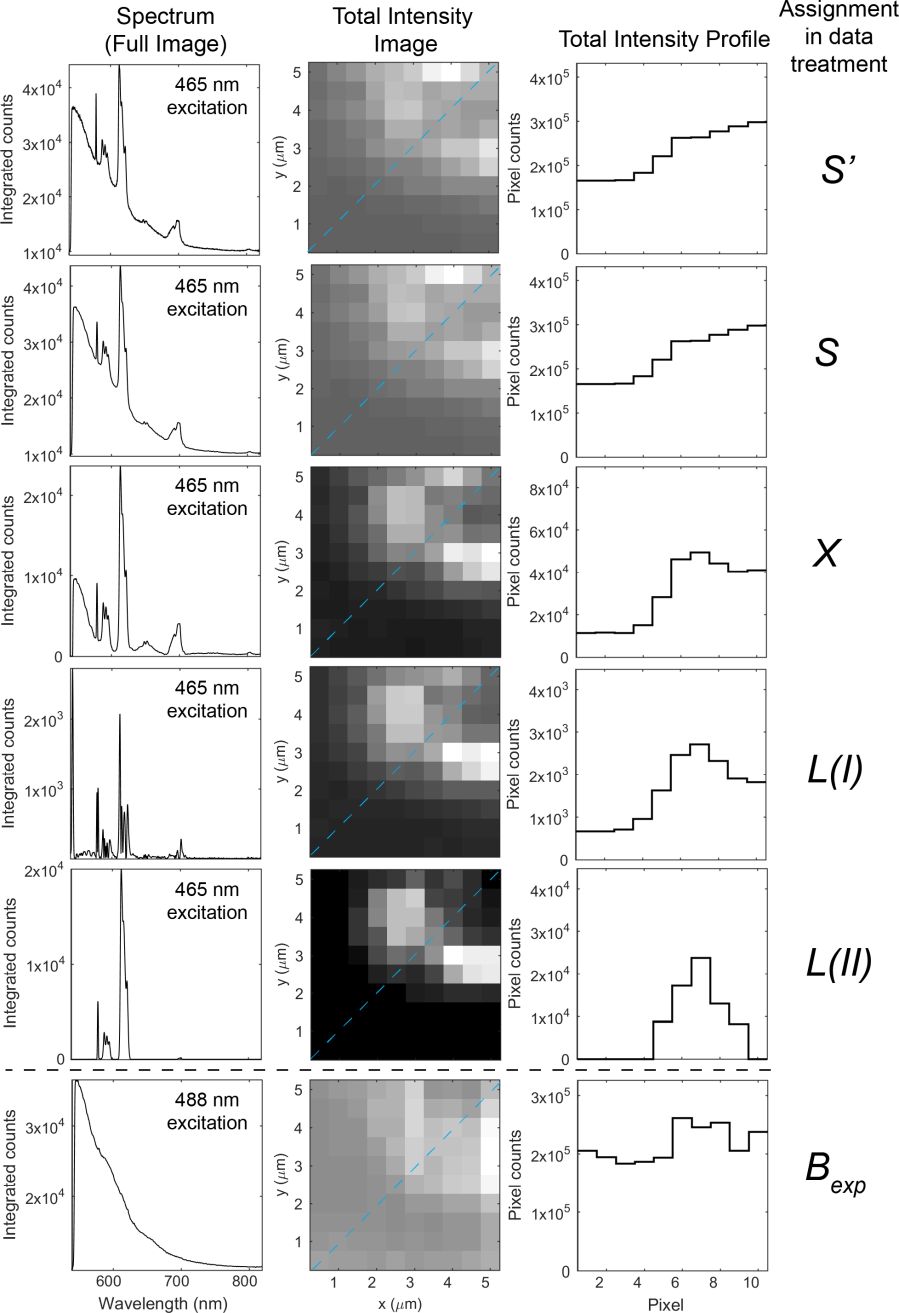
Description of the different signals used in the presented data treatment methods. Spectra created from the sum of the pixel-by-pixel treated spectra, total intensity images, and intensity profile plots corresponding to the blue dashed line in the total intensity images. The plotted signals arise from treatment of data recorded following excitation of a PVA thin film stained with F18, MitoTracker Red, ATTO647N, and LTA zeolites doped with europium(III) ions. Assignment of signals: *S’*, raw data. *S*, smoothed raw data; *X*, channel-by-channel background subtracted data with a built-in function; *L*, resolved data; *B*_*exp*_, experimentally determined background signal from another area of the sample.

To demonstrate our data treatment methods, we used data recorded on a scanning confocal fluorescence microscopy set-up that is capable of detecting the fluorescence of single molecules [39]. The microscope was used to record spectrally resolved images of a model system comprised of zeolites doped with lanthanide(III) ions in a polyvinyl alcohol (PVA) film on a glass cover slip. The model system is described in detail elsewhere [14]. In each image, the data recorded in each pixel were treated according to the scheme outlined in Fig 1. The effect of the data treatment is illustrated in Fig 2. The raw data (*S’*) constitutes the full emission spectrum recorded in each pixel, from which smoothed data (*S*) is created by using a Savitzky-Golay filter as implemented in the Matlab® software. In method I, the gradient (*Δ*) of the data *S* can be directly used to provide a sharp-feature-sensitive signal (*L(I)*), simply by resolving areas of the spectrum with high gradients. In method II such gradient is evaluated against a set of thresholds (*T1*, *T2*, and *T3*) and used to create the background signal (*B*) which serves as a baseline that is different for every pixel. This baseline is then used to resolve sharp features in the spectrum, creating a resolved signal (*L(II)*). In some cases, the background signal (*B*) may be compared to an experimentally determined background signal (*B*_*exp*_). The effect of the data treatment with method II is illustrated in Fig 2. A detailed walk-through of the data treatment methods I and II can be found in the methods section. Fig 1 includes a general standard background subtraction commonly found in any image treatment software, where the fluorescent signal in each pixel is reduced by a given threshold value (*T*), producing a background subtracted image (*X*). In order to construct the spectrally resolved images, a signal *L* is integrated in a part of the spectral window or over the whole spectral window.

Fig 2 shows spectra, images and intensity profiles of all the terms used in the data treatment. Note that the signals plotted in Fig 2 arise from treating the image pixel-by-pixel. Each signal is then extracted from spectrally resolved images of a PVA film homogeneously stained with 150 μM fluorescein-C18 (F18), 0.1 μM MitoTracker Red, and 0.1 μM ATTO647N together with zeolites containing an estimated amount of 50 mM europium(III) ions. The fluorophore emission emulates a very significant background signal (*B*) (mostly fluorescein when exciting at 465 nm or 488 nm), while the europium centered emission is the specific fluorescent signal. The raw data (*S’*) and the smoothed raw data (*S*) include detector noise, background and the specific fluorescent signal. Fig 2 includes a signal following a direct threshold-based background subtraction (*X*); a method commonly part of any imaging software and is included for comparison. Due to the complexity of the signal *S’*, this type of subtraction does not remove all the non-specific features of the background. This becomes obvious by comparing the experimentally determined background (*B*_*exp*_), the raw data (*S’*), and *X* (Fig 2).

Since we seek to fully resolve the fluorescent signal obtained from lanthanide centered emission we developed two different approaches presented as methods I and II. Fig 2 shows the result of the data treatment methods where the resolved data (*L*) recovers the sharp features of europium(III) centered emission.

Method I (*L(I)*) generates the contrast in the image directly from the gradient of the spectrum, while method II (*L(II)*), via the gradient, recovers the fluorescent signal originating in the europium(III) centered emission with a zero background. The fluorescent signal from method II can be quantified directly, and the calculated signal-to-background is infinite. Thus, the image has perfect contrast as shown in Fig 2.

### Details of the data treatment

**Fig 3:**
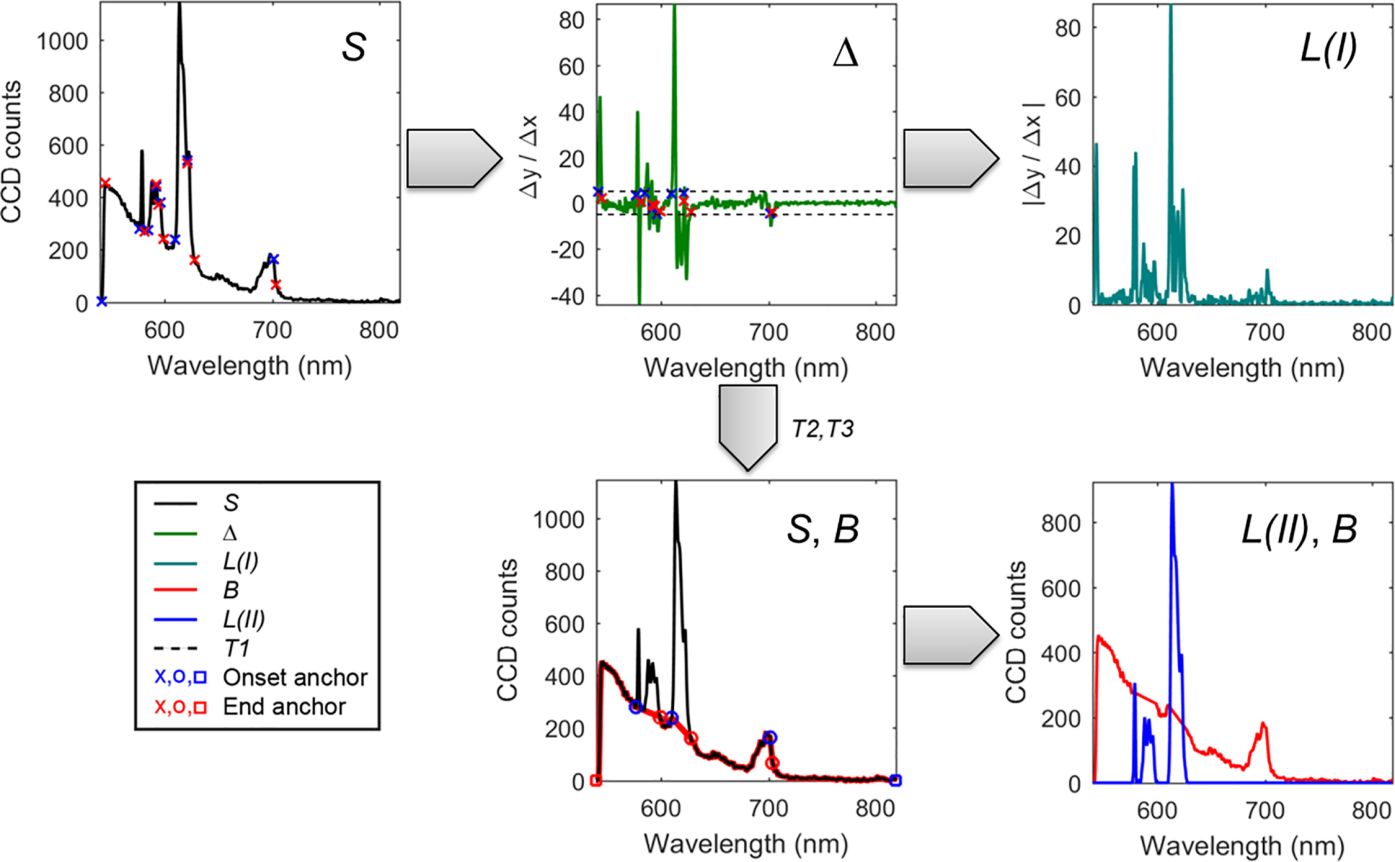
Walkthrough of methods I and II for resolving sharp emission bands in spectrally resolved data. In method I, the smoothed raw data (*S*) is differentiated, and the smoothed gradient (*Δ*) is used to calculate the signal *L(I)* as the absolute value of the gradient. In method II, the smoothed gradient (*Δ*) is compared with the threshold T1 to provide the first set of feature onset and end anchors. Using the thresholds *T2*, and *T3* the final anchor points are determined and the baseline (*B*) is defined. Subtracting the baseline (*B*) from the raw data resolves the fluorescent signal *L(II)* as the sharp emission bands. This baseline is identical to the background signal. The integration time of the spectrum shown is 1 second. All signals are extracted from a single bright pixel in the image shown in Fig 2.

We present two methods for automatically resolving the sharp emission features of lanthanides from the broad background fluorescence. Method I gives rise to an improved contrast, while method II goes a step further and resolves the fluorescent signal, see Fig 3, that shows data from a single pixel. Both methods follow the same initial steps: First, the narrow emission features are located using a simple *Δy/Δx* gradient, which uniquely identifies the onset points of each sharp peak. Method I sacrifices direct quantification of the fluorescent signal for speed, by generating the contrast directly by summing the absolute value of the gradient (*Δ*), see Fig 3. Method I reports this sum of gradient directly as contrast in the image, while method II resolves the fluorescent signal (*L*) by generating a baseline/background (*B*) from the gradient and subtracting it from the raw data while setting potentially appearing (in rare instances) negative values to zero (*L* = max(*S*–*B*, *0)*). Fig 3 shows that method II resolves both the fluorescent signal (*L*) and the background signal (*B*) using anchor points that are detected next to features in gradient (*Δ*) that are greater than a threshold (*T1*) in absolute value. Method II treats the data further in order to evaluate the distance of each feature by employing a second threshold (*T2*), here we use *T2* = 200 cm^-1^ as the minimum distance of the detected features to count all of the vibrational levels of one electronic transition of a lanthanide to one feature. A third threshold (*T3*) removes sharp features resulting from optical elements, such as optical filters. Finally, two anchor points are added so the baseline takes the limits of the spectrally resolved data into account (i.e., the beginning and end of spectrum). The background *B* is calculated using a simple definition: the values between consecutive onset-end anchors are linearly interpolated, while the values between consecutive end-onset anchors are equal to the data *S*. A walk-through and a graphical representation of this concept are provided in Fig 3.

Using our image analysis method, only photons emitted by molecular probes based on lanthanide luminescence with very narrow features will provide contrast. This method provides automatic background subtraction without previous knowledge on the lanthanide. A simplification by using predefined anchor points would be problematic in case of multi-labeling with different lanthanides. Matlab® scripts for automated data treatment according to methods I and II are included as supplementary information.

**Fig 4:**
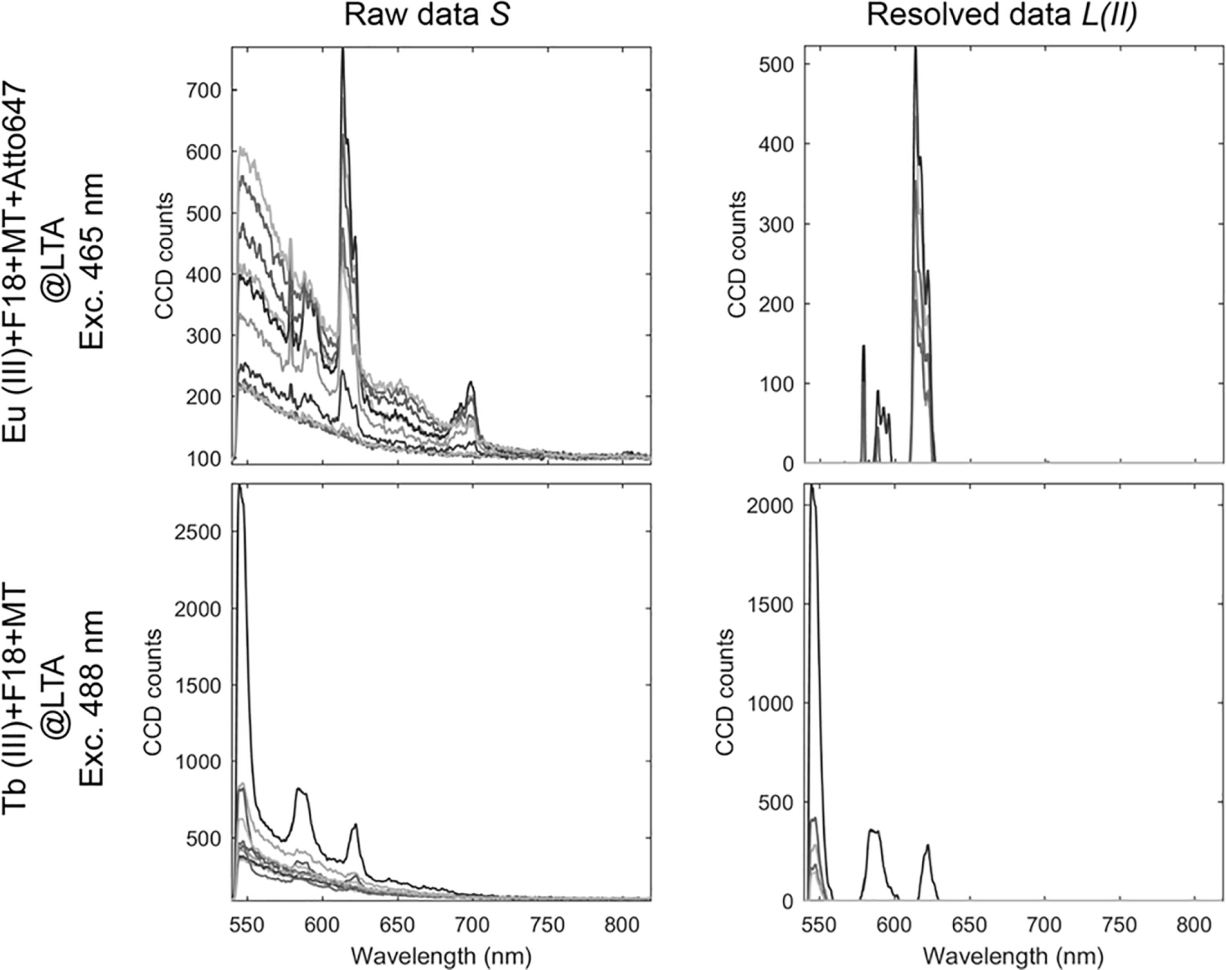
Smoothed spectra *S* and resolved spectra *L(II)* from individual pixels. Each spectrum corresponds to an individual pixel in an image of PVA thin film stained with F18, Mito Tracker Red, ATTO647N and LTA zeolites doped with europium(III) ions following 465 nm excitation and PVA thin film stained with F18, Mito Tracker Red and LTA zeolites doped with terbium(III) ions following 488 nm excitation.

Fig 4 shows the spectra recorded from individual pixels and demonstrates that the method works on a pixel-by-pixel basis. Moreover, Fig 4 illustrates that our data treatment method can resolve fluorescent signals of both europium(III) and terbium(III) ions with varying intensities. The variation in intensity can arise from differences in probe concentration or probe brightness. Fig 4 shows that all pixels where the lanthanide centered emission intensity rises above the background noise level are resolved. Thus, only the photons arising from the probes are used to form the image. Therefore, the resulting images are unique in two ways. Firstly, they have zero background. Secondly, the image is formed with full knowledge of the origin of the photons used to create the image. All photons represented as fluorescent signal in the image originate from the lanthanide based molecular probe.

Features from the lanthanide ions where the absolute value of the gradient is below the threshold *T1* are not resolved (Fig 4). This can occur if the signal from the lanthanide is too dim that it is comparable in intensity to the noise of the background. The onset from which the sharp features in the spectrum are resolved corresponds to a non-linear input-output relationship. Thus, the signal intensity does not scale linearly with probe concentration at low signal intensity. When the contribution of the lanthanide emission is sufficiently high for a band to be resolved as a sharp feature, the output *L* becomes linear in intensity and probe concentration. As the onset where a sharp feature is resolved varies, quantitative measurements should be performed using only a single sharp feature in the spectrum. The non-linear effects can be corrected by using specific anchor points in the software keyed to a specific band. One type of spectral filtering is described below.

### Multicolour images

**Fig 5:**
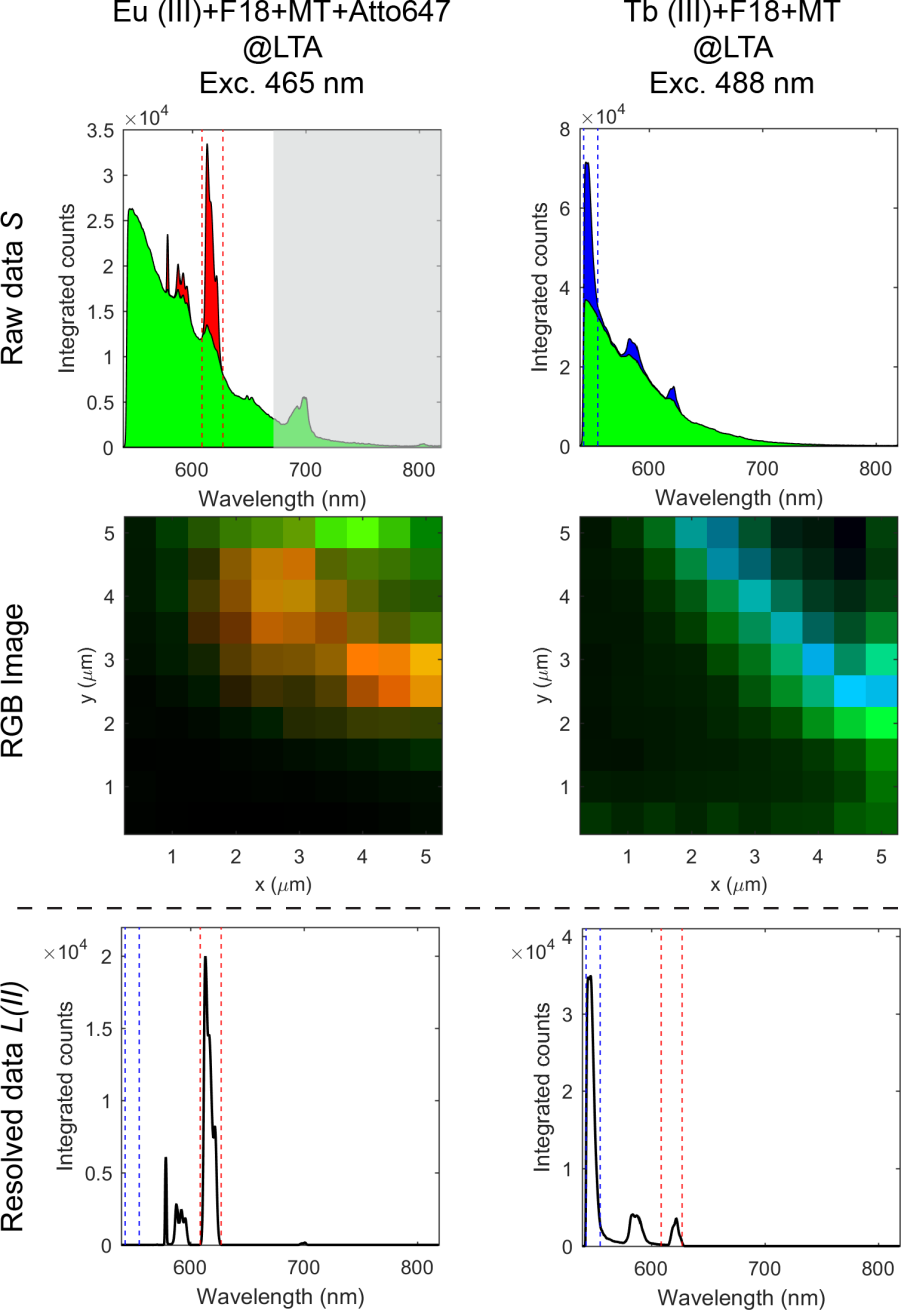
Images of multilabeled samples where method II is used to resolve the fluorescent signal from sharp emission bands and the background. Smoothed raw data *S* (integrated for all the pixels in the image) and RGB images where green color indicates the background signal *B* from F18, MitoTracker Red, and ATTO647N and red or blue color indicate the fluorescence *L(II)* from Eu(III) or Tb(III), respectively. The signal of the lanthanides *L(II)* is integrated inside the spectrally filtered band indicated for each image, whereas the signal *B* is integrated over the whole spectral region. In the case of Eu(III) the spectral region in gray color was not integrated, since the Eu(III) band in that region was not sharp enough to be resolved by method II. The R, G, and B images that form the merged RGB image are individually normalized in order to resolve the features detected in the background signal and from the lanthanide centered emission. In the bottom row, the resolved spectra *L* show that several lanthanide(III) ions can be imaged simultaneously if the fluorescent signals are integrated within a specific lanthanide band as illustrated by the dashed lines.

The obtained signals (*L*, *B*) can be keyed to a specific lanthanide by spectral gating. The emission lines of the individual lanthanide(III) ions can be compared to a barcode, and they will always be at a specific absolute and relative energies: a fact that can be included in the method when signals from multiple lanthanide based molecular probes are to be separated. In Figs 2 and 4, the method only operates by identifying sharp features, and the fluorescent signal included all the photons recorded in the features. However, Fig 5 shows how individual features can be selected by spectral gating in order to specifically differentiate between multiple lanthanides, here europium(III) and terbium(III) centered emissions are used.

## Discussion

While methods I and II can resolve any sharp features in spectrally resolved fluorescence microscopy, the emission lines arising from excitation of lanthanide(III) ions are ideal. Lanthanide based dyes can be used alone or in combination with organic dyes. In the latter case, the proposed methods can resolve the signal from both the organic and the lanthanide-based dyes, as illustrated in Fig 5. Thus, additional channels can be exploited in a multicolour experiment.

In contrast to fluorescence lifetime microscopy (FLIM) and time-gating,[12, 13] where the assumption is that only one probe emits photons with a given time constant or after a gate time, methods I and II use a unique signature of the probe to verify the origin of the emitted photons. The cost is that the intensity (number of photons) recorded in each pixel has to be large enough to resolve the signature(s) of the lanthanide based probes. FLIM has the same issues as a large number (>1.000) of photons has to be detected in order to resolve the fluorescence lifetime. If accurate lifetimes are to be recovered, more counts (>>10.000) are required and even then, lifetime distributions rather than distinct probe specific lifetimes are determined. Time-gating, while faster, cannot distinguish between long lived emitters of similar colour. Photon arrival time imaging (PArTI) can distinguish between emitters with emission lifetimes that differ by an order of magnitude, but do not do so with absolute contrast [13]. While the spectral features may vary in shape and lifetime the position is constant, which the presented data treatment methods use as a unique signature.

**Fig 6:**
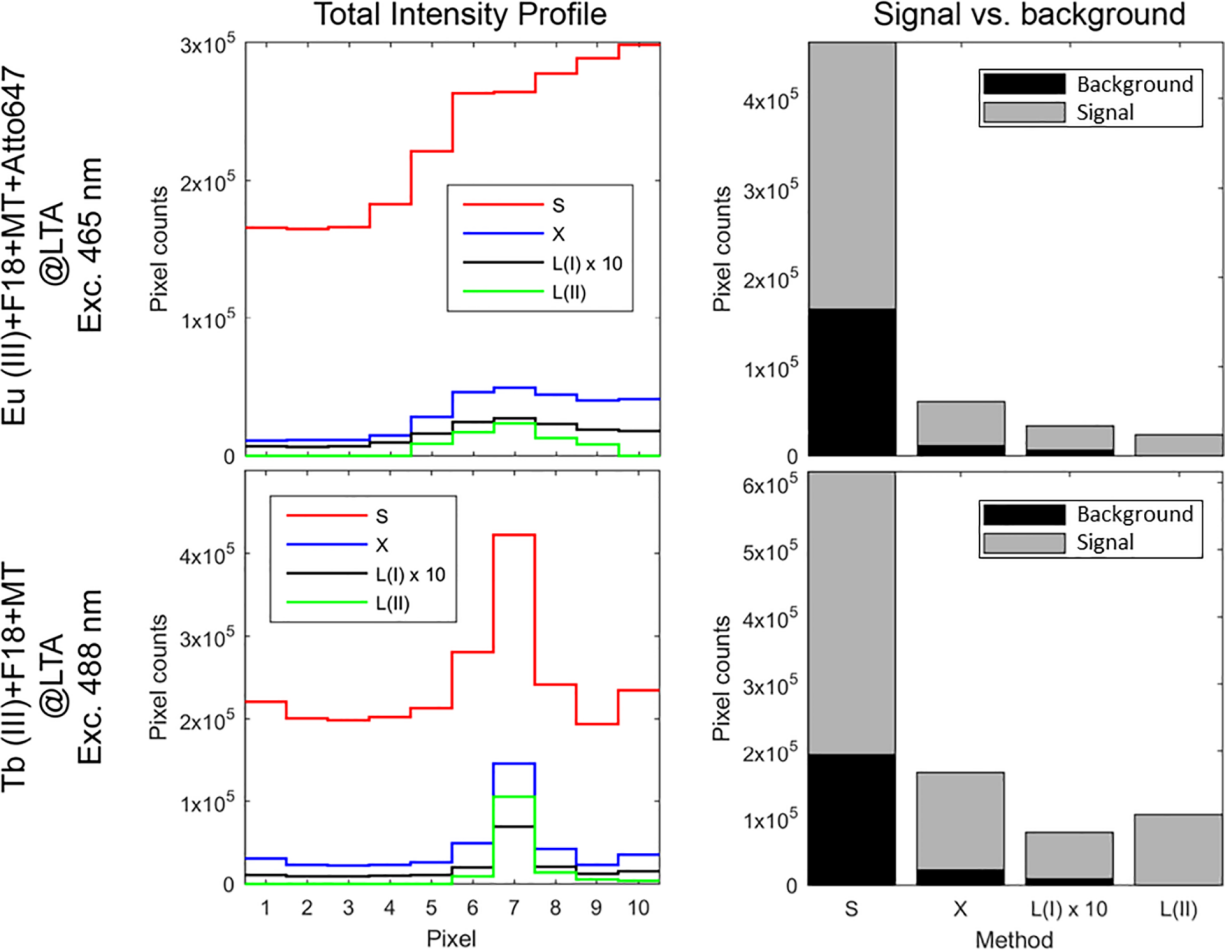
Comparison of signal and background of raw data and different background subtraction methods. Total fluorescent signal along the diagonal lines (Fig 2 for Eu, data for Tb not shown) and signal vs. background in images of PVA thin film stained with F18, Mito Tracker Red, ATTO647N and LTA zeolites doped with europium(III) ions following 465 nm excitation and Tb@LTA with F18 and MT following 488 nm excitation. The signal or background level corresponds to the maximum or minimum of the intensity profile, respectively.

It can be argued that due to the long emission lifetime of the europium(III) and terbium(III), high contrast can also be achieved using time-gating or circular polarized light [13, 15, 20]. The main difference is that the latter techniques require specialized equipment, while spectral imaging is already implemented in high-end commercial fluorescence microscopes, assuming the spectral resolution is sufficiently high in order to apply our method. Thus imaging using lanthanide based probes can readily benefit from absolute certainty as to whether an object in an image is due to the presence of the molecular probe or a strong background signal. While the total fluorescent signal is decreased, the signal-to-background level is drastically increased, see Fig 6. Using europium(III) as an example, the signal-to-background ratios for the signals *S*, *X*, *L(I)*, *L(II)* are 2.98·10^5^:1.65·10^5^, 4.95·10^4^:1.13·10^4^, 2.72·10^3^:6.7·10^2^ and 2.37·10^4^:0, respectively. In method I, the contrast can be improved by integrating the spectra at longer wavelengths than the filter onset, to avoid the contribution of the filter to the signal based on the gradient. This correction improves the method I contrast slightly, but absolute signal-to-background is achieved with method II.

Even though the spectral imaging technique as presented here is not capable of rapid image acquisition (integration times are up to 1 second per pixel), the two methods enable accurate discrimination of the lanthanide signal from the background signal and offer the possibility for more complex lanthanide multilabeling. The latter would cause difficulties in time gating using different lanthanides with similar decay times. We have used a home-build microscopy set-up, but other high-end modern microscopes with sufficient spectral imaging resolution should also be able to implement the data treatment methods outlined here. Furthermore, the better of the two methods allows the origin of the detected fluorescent signal to be assigned with full confidence; the detected photons arise only from emission of a specific molecular probe. Furthermore, as we use direct excitation of lanthanide(III) ions, no photobleaching occurs.

In summary, we have demonstrated the use of spectrally resolved fluorescence microscopy to identify features stained with lanthanide based molecular probes. We have presented two methods to resolve narrow emission lines resulting from lanthanide centered emission by using either the gradient (method I) or the photons within the narrow bands (method II) to provide contrast. While demonstrated using the very sharp features of lanthanide centered emission, the methods will resolve all features that are significantly sharper than the background fluorescence. We believe that method II is an excellent image analysis method that with absolute certainty can assign the origin of the photons used to generate an image of lanthanide(III) based luminescent probes.

## Supporting information

S1 File This file contains the Matlab® software main script for displaying images and spectra generated by the AutoBackgroundRemove_2p3.m function. To run the Matlab® program the *SpectralImaging_SharpBands_InfiniteContrast*.*m*, *AutoBackgroundRemove_2p3*.*m* and *Printer_subplot_Mig*.*m* files should be in the same local folder.
(M) Click here for additional data file.

S2 File This file contains the Matlab® function called in the main script to generate the spectrally resolved signal L(II). (M)

S3 File This file contains the Matlab® function called in the main script for plotting figures. (M)

S4 File This file describes the file format requirements of to run the Matlab® program. (DOCX)

S5 File This document describes the full lists of files included in S6 File and S7 File as well as the experimental details. (DOCX)

S6 File Image files. (ZIP)

S7 File Spectral data files. (ZIP)
