# Supplementary material for: Creating infinite contrast in fluorescence microscopy by using lanthanide centered emission
Source: PLoS One. 2017 Dec 13;12(12):e0189529. doi: 10.1371/journal.pone.0189529 (PMC5728579; doi:10.1371/journal.pone.0189529)
Supplement: S4 File — (DOCX) [file pone.0189529.s004.docx]

First, delete the Sx_file_ part of the filenames in the MATLAB script files.

The program *SpectralImaging_SharpBands_InfiniteContrast.m* needs to load a specific file format. Other file formats can be loaded by adding some modifications to the MATLAB code in the importing options. After modifying the number of headers and potentially ignored rows or columns, the important thing is that the program needs 2 variables:

- *J* is an *m*x*n* matrix containing the recorded counts from the detector. The rows *m* indicate the spatial pixel and the columns *n* indicate the spectral (CCD) pixel. The information of the spatial pixel is sorted by acquisition time. The code is set for left-right right-left reading mode of the piezo scanner, but can be modified if necessary.
- *J2* is an *n*x2 matrix where the first column contains the pixel number and the second column contains the wavelength value. In other words, a calibration file from the spectrometer. It should contain no headers or rows/columns to ignore.

The default file type imported in the program is a .txt file which contains a number of text headers and 2 rows with numbers that are ignored. The importing is done with the code:

Firstrow=3; mmax=10; nmax=10

I = importdata([datapath dataname '.txt']);

I2 = importdata([calibrationpath calibrationname '.txt']);

J=I.data(firstrow:mmax*nmax+firstrow-1,1:end);

J2=I2(:,2);

Where firstrow=3 indicates that the 2 rows are ignored, mmax is the number of rows of the image and nmax is the number of columns of the image. The total number of spatial pixels is mmax*nmax.

If there are no headers in the data file with the intensity values, J would be no longer a structure array and the code should be changed to:

J=I (firstrow:mmax*nmax+firstrow-1,1:end);
