# Supplementary material for: Creating infinite contrast in fluorescence microscopy by using lanthanide centered emission
Source: PLoS One. 2017 Dec 13;12(12):e0189529. doi: 10.1371/journal.pone.0189529 (PMC5728579; doi:10.1371/journal.pone.0189529)
Supplement: S5 File — (DOCX) [file pone.0189529.s005.docx]

Supporting information

# Creating infinite contrast in fluorescence microscopy by using lanthanide centered emission

Miguel R. Carro-Temboury,^1^ Riikka Arppe,^1^ Casper Hempel,^2*^ Tom Vosch^1*^ and Thomas Just Sørensen^1*^

^1^Nano-Science Center & Department of Chemistry, University of Copenhagen, Copenhagen, Denmark

^2^ Dept of Micro- and Nanotechnology, Technical University of Denmark, Kgs Lyngby, Denmark

# Table of contents

[Table of contents 1](#_Toc495399054)

[Content of the SI: 2](#_Toc495399055)

[Samples: 2](#_Toc495399056)

[Imaging the samples: 2](#_Toc495399057)

[Image files in S2_file.zip: 3](#_Toc495399058)

[Spectral data in S3_file.zip: 4](#_Toc495399059)

# Content of the SI:

The SI contains this document, files with image data, and files with spectral data. All data are from the seven samples in the list below. Additional data has been added per request of the reviewers; this data is added at the end of this document.

# Samples:

S1: 100 µg/ml F18, Tb-zeolites, Eu-zeolites; in 3% (w/v) PVA

S2: 0.1 µM MitoTracker Red, Tb-zeolites, Eu-zeolites; in 3% (w/v) PVA

S3: 0.1 µM ATTO647N, Tb-zeolites, Eu-zeolites, EuTb-zeolites; in 3% (w/v) PVA

S4: 100 µg/ml F18, 0.1 µM MitoTracker Red, Tb-zeolites, Eu-zeolites; in 3% (w/v) PVA

S5: 0.1 µM MitoTracker Red, 0.1 µM ATTO647N, Tb-zeolites, Eu-zeolites; in 3% (w/v) PVA

S6: 100 µg/ml F18, 0.1 µM ATTO647N, Tb-zeolites, Eu-zeolites; in 3% (w/v) PVA

S7: 100 µg/ml F18, 0.1 µM MitoTracker Red, 0.1 µM ATTO647N, Tb-zeolites, Eu-zeolites; in 3% (w/v) PVA

# Imaging the samples:

A single zeolite was located and it surroundings were imaged using the four excitation wavelengths (465 nm, 488 nm, 560 nm and 633 nm) so that each excitation wavelength was used in separate corners of the zeolite (Figure S1). This was done in order to minimize the bleaching of the dyes.


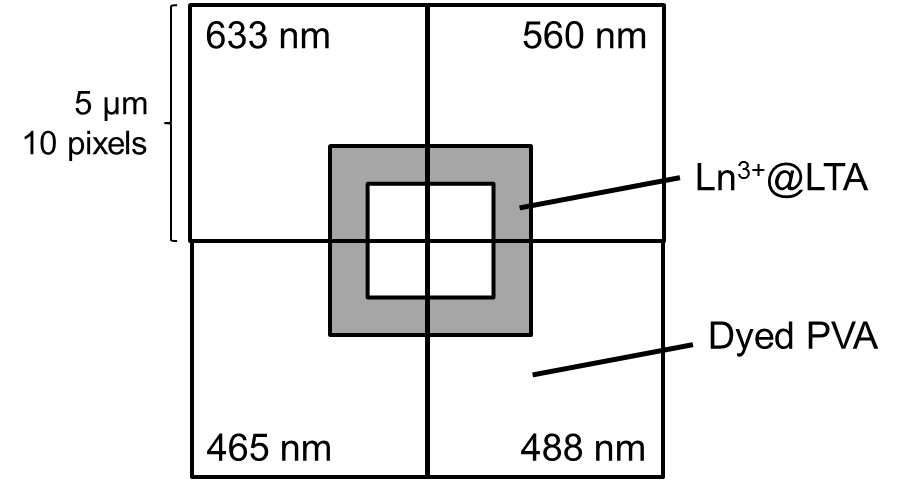


**Figure S1. Schematic of the areas used for imaging one zeolite using the four excitation wavelengths.**

# Image files in S7_file.zip:

The file names show the sample number, the lanthanide dopant, the excitation wavelength, and the location of the image in respect to the zeolite in the middle, respectively (see Figure S1).

## S1:

- S1_Tb_465_lowleft
- S1_Tb_488_lowright
- S1_Tb_560_topright
- S1_Tb_633_topleft
- S1_Eu_465_lowleft
- S1_Eu_488_lowright
- S1_Eu_560_topright
- S1_Eu_633_topleft

## S2:

- S2_Tb_465_lowleft
- S2_Tb_488_lowright
- S2_Tb_560_topright
- S2_Tb_633_topleft
- S2_Eu_465_lowleft
- S2_Eu_488_lowright
- S2_Eu_560_topright
- S2_Eu_633_topleft

## S3:

- S3_EuTb_560_topright
- S3_EuTb_633_topleft
- S3_Tb_465_lowleft
- S3_Tb_488_lowright
- S3_Tb_560_topright
- S3_Tb_633_topleft
- S3_Eu_465_lowleft
- S3_Eu_488_lowright
- S3_Eu_560_topright
- S3_Eu_633_topleft

## S4:

- S4_Tb_465_lowleft
- S4_Tb_488_lowright
- S4_Tb_560_topright
- S4_Tb_633_topleft
- S4_Eu_465_lowleft
- S4_Eu_488_lowright
- S4_Eu_560_topright
- S4_Eu_633_topleft

## S5:

- S5_Tb_465_lowleft
- S5_Tb_488_lowright
- S5_Tb_560_topright
- S5_Tb_633_topleft
- S5_Eu_465_lowleft
- S5_Eu_488_lowright
- S5_Eu_560_topright
- S5_Eu_633_topleft

## S6:

- S6_Tb_465_lowleft
- S6_Tb_488_lowright
- S6_Tb_560_topright
- S6_Tb_633_topleft
- S6_Eu_465_lowleft
- S6_Eu_488_lowright
- S6_Eu_560_topright
- S6_Eu_633_topleft

## S7:

- S7_Tb_465_lowleft
- S7_Tb_488_lowleft
- S7_Tb_560_topright
- S7_Tb_633_topleft
- S7_Eu_465_lowleft
- S7_Eu_488_lowright
- S7_Eu_560_topright
- S7_Eu_633_topleft

## Empty zeolites_PVA no dyes:

- EmptyZ_PVA no dyes_465_lowleft
- EmptyZ_PVA no dyes_488_lowright
- EmptyZ_PVA no dyes_560_topright
- EmptyZ_PVA no dyes_633_topleft

# Spectral data in S8_file.zip:

Spectra were acquired from both the PVA-background and from the brightest pixel on top of the zeolite. The file names indicate the sample number, the lanthanide dopant, the excitation wavelength, and the pixel in the corresponding image.

## S1_PVA:

- S1_Tb_465_x1y2
- S1_Tb_488_x10y1
- S1_Tb_560_x10y10
- S1_Tb_633_x1y2

## S1_Zeolite:

- S1_Tb_465_x4y10
- S1_Tb_488_x7y10
- S1_Tb_560_x7y1
- S1_Tb_633_x8y4
- S1_Eu_465_x5y10
- S1_Eu_488_x9y10
- S1_Eu_560_x4y3
- S1_Eu_633_x10y8

## S2_PVA:

- S2_Eu_465_x10y1
- S2_Eu_488_x10y10
- S2_Eu_560_x9y10
- S2_Eu_633_x1y10

## S2_Zeolite:

- S2_Tb_465_x7y8
- S2_Tb_488_x7y8
- S2_Tb_560_x5y6
- S2_Tb_633_x5y4
- S2_Eu_465_x10y9
- S2_Eu_488_x5y10
- S2_Eu_560_x3y4
- S2_Eu_633_x10y5

## S3_PVA:

- S3_Eu_465_x1y1
- S3_Eu_488_x8y2
- S3_EuTb_560_x5y10
- S3_EuTb_633_x1y10

## S3_Zeolite:

- S3_Tb_465_x6y10
- S3_Tb_488_x1y10
- S3_Tb_560_x2y4
- S3_Tb_633_x9y4
- S3_Eu_465_x5y9
- S3_Eu_488_x3y5
- S3_Eu_560_x2y8
- S3_Eu_633_x8y5

## S4_PVA:

- S4_Tb_465_x1y1
- S4_Tb_488_x10y1
- S4_Tb_560_x8y10
- S4_Tb_633_x10y10

## S4_Zeolite:

- S4_Tb_465_x5y10
- S4_Tb_488_x6y10
- S4_Tb_560_x4y4
- S4_Tb_633_x7y3
- S4_Eu_465_x5y10
- S4_Eu_488_x1y9
- S4_Eu_560_x1y6
- S4_Eu_633_x7y3

## S5_PVA:

- S5_Tb_465_x10y1
- S5_Tb_488_x10y1
- S5_Tb_560_x10y10
- S5_Tb_633_x1y10

## S5_Zeolite:

- S5_Tb_465_x6y7
- S5_Tb_488_x1y5
- S5_Tb_560_x1y5
- S5_Tb_633_x10y5
- S5_Eu_465_x4y7
- S5_Eu_488_x1y6
- S5_Eu_560_x1y4
- S5Eu_633_x10y5

## S6_PVA:

- S6_Eu_465_x1y10
- S6_Eu_488_x10y10
- S6_Eu_560_x10y10
- S6_Eu_633_x1y10

## S6_Zeolite:

- S6_Tb_465_x7y10
- S6_Tb_488_x8y10
- S6_Tb_560_x3y5
- S6_Tb_633_x7y1
- S6_Eu_465_x7y10
- S6_Eu_488_x4y10
- S6_Eu_560_x2y3
- S6_Eu_633_x10y3

## S7_PVA:

- S7_Tb_465_x1y1
- S7_Tb_488_x10y1
- S7_Eu_560_x1y10
- S7_Eu_633_x1y10

## S7_Zeolite:

- S7_Tb_465_x10y7
- S7_Tb_488_x10y7
- S7_Tb_560_x1y2
- S7_Tb_633_x8y4
- S7_Eu_465_x8y10
- S7_Eu_488_x4y10
- S7_Eu_560_x1y6
- S7_Eu_633_x10y10

## PVA without dyes:

- 465_x1y2
- 488_x10y1
- 560_x7y10
- 633_x1y10

## Empty zeolites:

- 465_x8y10
- 488_x5y10
- 560_x4y4
- 633_x7y3
